# Mechanistic Insights into Anion-Induced Electrochromism of Ru(II)-Based Metallo-Supramolecular Polymer

**DOI:** 10.3390/polym15244735

**Published:** 2023-12-18

**Authors:** Xiaofang Fu, Zhuohui Zhang, Zhenhu Cao, Alexandr Alexandrovich Rogachev, Maxim Anatolievich Yarmolenko, Tao Chen, Hongtao Cao, Hongliang Zhang

**Affiliations:** 1 Laboratory of Advanced Nano Materials and Devices, Ningbo Institute of Materials Technology and Engineering, Chinese Academy of Sciences, Ningbo 315201, Chinah_cao@nimte.ac.cn (H.C.); 2 Center of Materials Science and Optoelectronics Engineering, University of Chinese Academy of Sciences, Beijing 100049, China; 3 Nano Science and Technology Institute, University of Science and Technology of China, Suzhou 215123, China; 4 Ningbo Mi Ruo Electronic Technology Co., Ltd., Ningbo 315203, China; 5 Optical Anisotropic Films Laboratory, Institute of Chemistry of New Materials of the National Academy of Sciences of Belarus, 220141 Minsk, Belarus; 6 Department of Radiophysics and Electronics, Francisk Skorina Gomel State University, 104, Sovetskaya Street, 246019 Gomel, Belarus

**Keywords:** electrochromic film, Ru(II)-based metallo-supramolecular polymer, kinetics of ion transport, degradation mechanism, anion-induced electrochromism

## Abstract

The metallo-supramolecular polymer (MSP) is considered one of the most promising electrodes for electrochromic devices due to its intrinsically stable redox properties. Nevertheless, despite extensive work focusing on improving the electrochromic and electrochemical properties of MSPs, little experimental evidence exists from in-depth investigations on the anion-induced electrochromism of MSPs. Herein, Ru-based metallo-supramolecular polymer (polyRu) films with excellent electrochromic performance were fabricated through a novel electrochemical deposition method, and the electrochromic mechanism was further understood. The polyRu films possess fast reaction kinetics with a short switching time of 4.0 s (colored) and 2.8 s (bleached) and highly reversible redox properties due to the resulting impacts on the capacitive behaviors (containing surface, near-surface and intercalation pseudo-capacitance) of the perchlorate ions in the electrochromic process. Moreover, the electrochromic degradation of the polyRu films is considered to stem from the numerous nanopores in the film induced by ClO_4_^−^ transport and the exchange of counter anions from Cl^−^ to ClO_4_^−^. In addition, a physical model, revealing the transport of conduction ions and the evolution of the structure and properties of the polyRu film during the electrochromic process, is presented. It is observed that the charge balance of Ru^3+^ and Ru^2+^, achieved through the adsorption/desorption of ClO_4_^−^ on the film, provides electrochromic and electrochemical reversibility to the polyRu film under positive/negative bias. Correspondingly, a transformation from polyRu·(Cl^−^)_2n_ to polyRu·(ClO_4_^−^)_x_(Cl^−^)_2n−x_ in the polyRu film is induced by a counter anion exchange from Cl^−^ to ClO_4_^−^. Revealing the detailed perchlorate ion transfer kinetics and electrochromic mechanism in this film can offer new insights into the application of metallo-supramolecular polymers in electrochromic devices.

## 1. Introduction

A color change in materials induced by external stimuli (including gas, light, heat and electricity) can be referred to as chromism [1,2,3,4]. Among these changes, color modulation (changes in transmittance, absorbance and reflectance) by an applied external voltage is defined as electrochromism [5,6]. Electrochromic (EC) materials have received significant attention for their various applications in smart windows [7,8], antiglare rear-view mirrors [9], information displays [10], etc. Diverse EC materials have been exploited so far, including transition metal oxides [11,12,13], transition metal complexes [14,15,16], organic small molecules [17,18], organic conducting polymers [19,20] and metallo-supramolecular polymers [21,22,23]. In particular, metallo-supramolecular polymers (MSPs) are recently developed electrochromic materials with superior electrochromic performance and stable redox properties [5,21]. The chelating effects of metallic centers with organic ligands represent a driving force for the formation of metallo-supramolecular polymers [24]. MSPs show intense color changes owing to the metal-to-ligand charge transfer (MLCT) absorption [22,25]. Most of the previous research on MSPs has focused on developing novel methods of preparation to improve their microstructures and electrochromic properties, including the synthesis of metallo-supramolecular polymers self-assembled from various pyridine ring-substituted bisterpyridines and metal ions [26] and high-performance asymmetric supercapacitors with superb cyclic stability for 10,000 cycles [27]. Additionally, Hsiao et al. have proposed a Ru(II)/Fe(II)-based heterometallo-supramolecular polymer as the working electrode in electrochromic devices (ECDs). ECDs have been demonstrated to exhibit excellent electrochromic performance with remarkable coloration efficiency of 525.1 cm^2^ C^−1^, high transmittance modulation of 52.7% and a fast response rate of less than 0.5 s at 503 nm [22]. Supported by the electrostatic spray deposition method, Cai et al. have implemented a metallo-supramolecular polymer film that operates with unprecedented performance of large optical modulation, exceptional coloration efficiency, a fast response time and robust stability [23]. Recent studies reported by Roy et al. support that the introduction of sodium polystyrene sulfonate (PSS) in Fe(II)-based metallo-supramolecular polymers (polyFe) results in high-performance electrochromic devices [28]. Nevertheless, even though a lot of efforts have been made in recent years, the precise microscopic mechanisms regulating the electrochromic process of MSP films remain unclear. Recently, M. Eguchi et al. tried to establish a physical model of charge transfer in an MSP film during the electrochemical process, in the hope of revealing its electrochemical reaction kinetics [29]. One of the key extrinsic factors is the transport of conduction anions (ClO_4_^−^), which are largely used for defects and damage on the surface of Cu-based metal-supramolecular polymer films during the electrochemical process [24]. Unfortunately, very little is known about the mechanism of anion-induced electrochromism in MSP films, which severely limits our ability to design highly stable and robust electrochromic or electrochemical devices.

**Figure 1:**
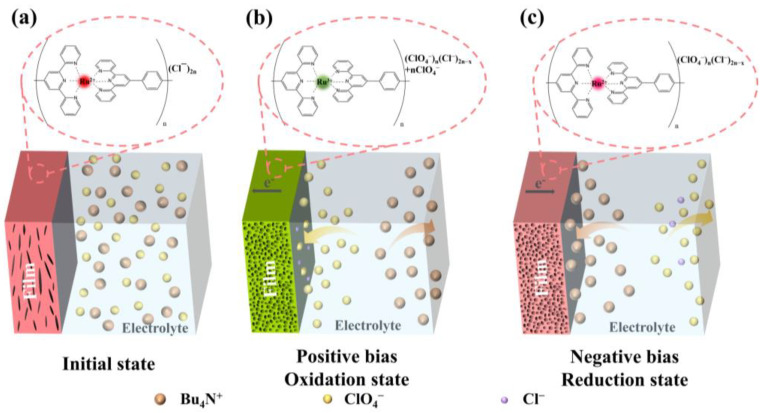
A schematic diagram of the ionic kinetic behavior and the structural evolution of the initial polyRu film (**a**) and the polyRu film under positive (**b**) and negative (**c**) bias.

Herein, a novel electrochemical deposition method which combines both chemical and electrochemical processes for the deposition of Ru(II)-based metallo-supramolecular polymer (polyRu) films is presented, based on a precursor solution formed by dissolving the synthesized polyRu powder into a mixture of methanol and dimethylformamide. We guide the reader through an overall assessment of the electrochemically deposited polyRu films after presenting the obtained electrochemical and electrochromic properties. The main purpose of this article is to elucidate the mechanism of anion-induced electrochromism in MSP films in a non-intercalating Bu_4_NClO_4_-MeCN electrolyte on the basis of ex situ and in situ characterization for the electrochemically deposited MSP films, as shown in Figure 1.

## 2. Experiment

### 2.1. Materials

Acetone (99.5%) was obtained from Sinopharm Chemical Reagent Co., Ltd. (Shanghai, China), while terephthalaldehyde (98%), 2-Acetylpyridine (98%), potassium hydroxide (KOH, ACS), ammonium acetate (AR), ruthenium chloride trihydrate (RuCl_3_·H_2_O, 98%), methanol (MeOH, 99.5%), ethanol (EtOH, ACS), tetrahydrofuran (THF, 99.5%), acetic acid (99.5%), dimethyl sulfoxide (DMSO, 99%) and ethylene glycol (98%) were received from Aladdin (Shanghai, China). All solvents and reagents were used without further purification.

### 2.2. Synthesis

#### 2.2.1. The Preparation of PolyRu Powder

The polyRu polymer powder was synthesized according to the published literature [27]. Equimolar amounts of 4′,4″-(1,4-phenylene) bis-2,2′:6′,2″-terpyridine and RuCl_2_(DMSO)_4_ were refluxed in nitrogen-saturated ethylene glycol for 24 h, with their synthesis described in detail elsewhere [26]. After the reaction solution was cooled to room temperature, THF was added until the solution was colorless. Red precipitates were obtained through centrifugation after standing. The red precipitates were washed with THF three times, and then, the precipitated products were dried under vacuum to obtain polyRu powder with a yield of about 90%.

#### 2.2.2. The Preparation of PolyRu Films

The Ru(II)-based metallo-supramolecular polymer (polyRu) films were prepared through two-electrode electrochemical deposition. ITO-coated glass and a platinum sheet were used as the working electrode and the counter electrode, respectively. We dissolved 100 mg polyRu powder in 50 mL methanol:dimethylformamide 1:1 mixed solution and stirred to obtain the precursor solution for electrochemical deposition. Then, the polyRu films were deposited at a constant potential of 8.0 V for 8 min using a potentiostatistical method. Subsequently, these samples were dried at 60 °C for 6 h in air.

### 2.3. Measurements

The cross-sectional morphologies of the polyRu films were characterized using a field emission scanning electron microscope (FESEM, S4800, Hitachi, Japan). The optical spectroscopy measurements were performed via UV-vis spectroscopy (Perkin–Elmer, Lambda 950, Waltham, MA, USA). The electrochemical properties of the polyRu films were analyzed on an electrochemical workstation (CHI660D, Chen Hua, Shanghai, China). CV measurements of the polyRu films were carried out in a standard three-electrode system, in which the polyRu film in ITO glass served as a working electrode, the 0.1 M acetonitrile solution of Bu_4_NClO_4_ served as an electrolyte solution, and a platinum sheet served as the counter electrode. The electrochemical impedance spectra (EIS) were recorded using an electrochemical workstation (Zennium, IM6, Kronach, German) at a frequency range of 100 mHz–100 kHz.

## 3. Results and Discussion

**Figure 2:**
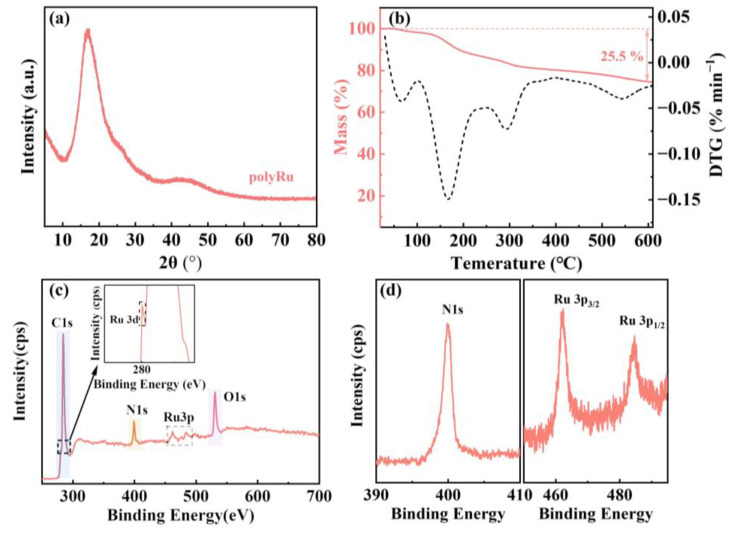
(**a**) the XRD pattern of the polyRu powder; (**b**) the thermogravimetric curve of the polyRu powder in nitrogen at a scan rate of 10 °C min^−1^ from room temperature to 610 °C; (**c**) the full XPS spectrum of the polyRu powder; (**d**) the fine XPS spectra of N 1s and Ru 3p in the polyRu powder.

XRD, TGA, XPS and TEM measurements were performed to confirm the synthesis of the polyRu MSP powder. The absence of diffraction peaks in Figure 2a suggests that the polyRu powder has amorphous characteristics. This can be attributed to the preparation process of the polyRu powder. When tetrahydrofuran was added to the mixed solution after the reflux reaction, it led to the formation of flocculent precipitation without a regular crystal structure. Combined with the TG and DTG curves in thermogravimetric analysis (Figure 2b), the polyRu powder has slight mass loss below 100 °C, corresponding to the residual water molecules and solvent molecules. The subsequent mass decline is caused by the slow decomposition of the metal–organic framework. From room temperature to 610 °C, the cumulative weight loss of the MSP is 25.5%, indicating that the synthesized polyRu powder has good thermal stability. As shown in Figure 2c,d, the characteristic peaks of C 1s, N 1s and O 1s occur at 284, 399 and 531 eV. Additionally, peaks corresponding to Ru 3d, Ru 3p3/2 and Ru 3p1/2 are detected at 280, 462 and 483 eV, demonstrating the presence of Ru in this polymer. Based on these experimental intensity ratios, the N/Ru atomic ratio is calculated to be 6.5:1, indicating that the metal is bound to the ligand in a hexagonal form. The analysis of the TEM image (Figure S1) showed no crystallite orientations, providing evidence for the amorphous nature of the synthesized polyRu powder, conforming the XRD result. In summary, we synthesized a polyRu powder with a metal–polymer hexagonal amorphous structure, which allows the powder to remain stable in a wide temperature range of room temperature to 610 °C.

**Figure 3:**
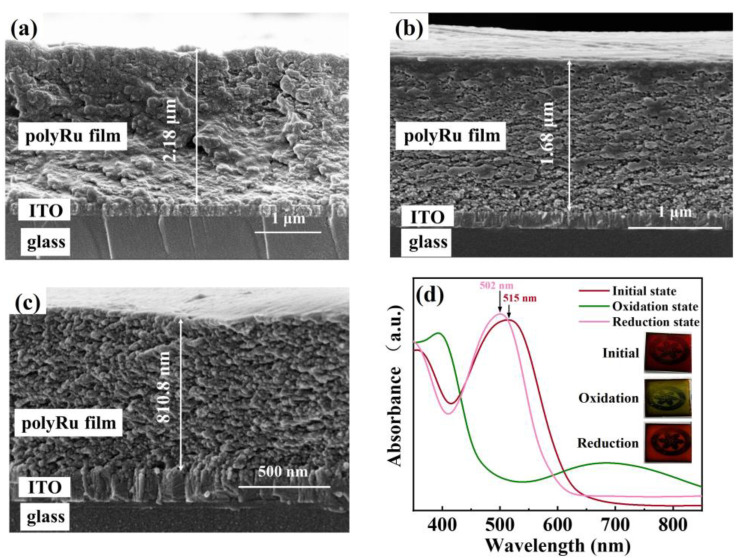
(**a**) Cross-sectional images of the initial polyRu film and the polyRu films treated by CV after (**b**) 100 and (**c**) 1000 cycles; (**d**) UV-vis spectra of the initial polyRu film and the polyRu films in oxidation and reduction states. Inset: digital photographs of the polyRu films in various states.

Figure 3a–c show the cross-sectional images obtained by the scanning electron microscope (SEM) for the as-deposited polyRu film and the polyRu films after the CV (for 100 and 1000 cycles) treatment process in the non-intercalating Bu_4_NClO_4_-MeCN electrolyte, respectively. As can be seen, the as-deposited polyRu film without obvious pores is relatively homogeneous and compact, and exhibits perfect adherence to the ITO substrate. The thickness of the as-deposited polyRu film is estimated to be approximately 2.18 μm. There are many irregularly distributed nanopores in the polyRu film treated after cyclic voltammetry in the Bu_4_NClO_4_-MeCN electrolyte. Such nanopores are probably caused by the shedding of the polyRu monomer or the oligomer outside the polyRu film, which does not connect well with polymer matrix due to the transport of the ClO_4_^−^ anion in the film [24]. As the number of cycles further increases, the number and density of nanopores increase, and the film thickness gradually decreases (from 1.68 μm to 810.8 nm). The resulting degradation of the electrochromic performance of the polyRu film is observed (Figure S2). This degradation is also reflected in the long-term durability measurement, where the capacity of the polyRu electrode material decreases as the number of cycles increases (Figure S3). The color of the electrolyte changes from clear to yellow (Figure S4), indicating the polymer’s shedding into the electrolyte during the long-term durability test. In addition, the color of the polyRu film changes from rose red in the initial state to green in the oxidation state, and to dark red in the reduction state (close to the initial color), as shown in the inset of Figure 3d. To address this issue of the transport of ClO_4_^−^ anions being disadvantageous for the performance of polyRu films, some possible strategies have been proposed. Firstly, enhancing the interaction between the metal ions and ligands can help to improve the stability of the polymer chain in the electrolyte, as demonstrated in the previous report [30]. Alternatively, creating a looser structure in electrochromic materials can facilitate the transfer or migration of ion carriers across the film, thereby improving the impact of anion transport to some extent [31]. It is well known that the color of the film depends on its absorption. As far as the polyRu film is concerned, the MLCT absorption from the d-electron of Ru(II) to the LUMO (the lowest unoccupied molecular orbital) of the π-conjugated ligand is critical. The strong MLCT absorption band of 515 nm in the initial state is shifted to 502 nm in the reduction state of the polyRu film, as shown in Figure 3d. This shift is probably attributed to the exchange of the counter anion from Cl^−^ to ClO_4_^−^ in the film during the electrochemical process, consistent with previous studies [25,32].

The transport and action mechanism of the counter anion (ClO_4_^−^) from the supporting electrolyte in the electrochromic process of the Ru(II)-based metallo-supramolecular polymer (polyRu) film is shown in Figure 1. Figure 1a illustrates the initial state of the film. The polyRu film shows dark red owing to metal-to-ligand charge transfer (MLCT) absorption. The cations and anions are uniformly distributed in the acetonitrile solvent, and the ions in the electrolyte may spontaneously wet the electrode material due to the concentration gradient. With a positive bias applied to the polyRu film, as shown in Figure 1b, electrons are extracted from the polyRu film, and ClO_4_^−^ anions in the electrolyte are attracted towards the electrode surface due to the electric field. At the same time, Ru^2+^ ions present in the polyRu film undergo oxidation and are converted into Ru^3+^ ions. As a result, ClO_4_^−^ (nClO_4_^−^) anions at the electrode/electrolyte interface are absorbed into the polyRu film to neutralize the change in the charge of the metal ions (from Ru^2+^ to Ru^3+^), accompanied by an exchange of the counter anion from Cl^−^ to ClO_4_^−^ ((ClO_4_^−^)_x_) in the film [25]. Therefore, the polyRu film changes from dark red in the as-deposited state to dark green in the oxidation state. Simultaneously, the film material exhibits numerous defects and damage due to the exchange of counter anions, which is likely to be one of the crucial reasons for the degradation in the electrochromic properties of the polyRu film. Conversely, when a negative voltage is applied to the polyRu film, as shown in Figure 1c, electrons are injected into the polyRu film, and the oxidated form of the polyRu film (Ru^3+^) becomes reduced to Ru^2+^. Simultaneously, anions ClO_4_^−^ (nClO_4_^−^) previously absorbed in the film are desorbed, and then, repelled away from the electrode surface with the influence of the electric field. Conversely, the color of the polyRu film changes from dark green in the oxidation state to rose red in the reduction state, instead of dark red in the as-deposited state, due to the change in counter anions in the film ((ClO_4_^−^)_x_(Cl^−^)_2n−x_) [33,34].

**Figure 4:**
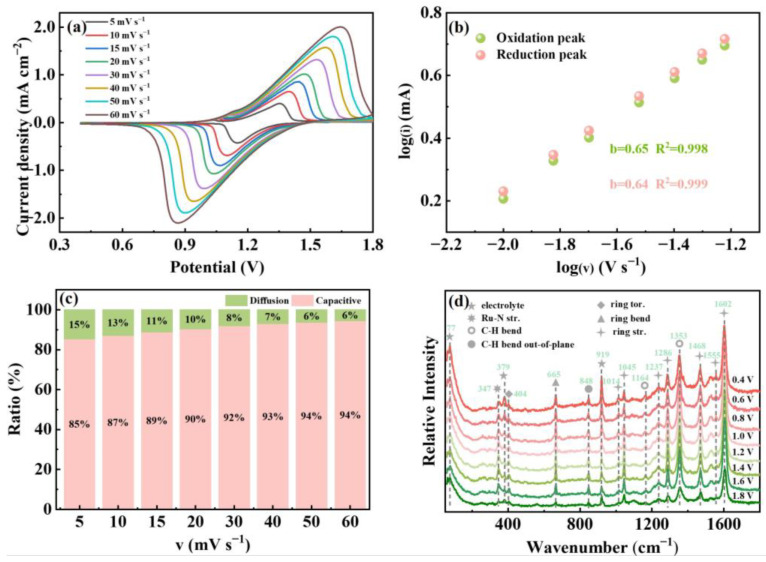
(**a**) CV curves of the polyRu film at scan rates of 5, 10, 15, 20, 30, 40, 50 and 60 mV s^−1^, respectively. (**b**) Powder law dependence of the peak current versus the scan rate for the polyRu film in oxidation and reduction states. (**c**) The contribution of capacitive behavior at different scan rates of the polyRu film in the electrochemical process. (**d**) In situ Raman spectra of the polyRu film at various potentials.

To understand the ClO_4_^−^ anion transport process in the polyRu film, the cyclic voltammetry curves were measured at different scan rates ranging from 5 mV s^−1^ to 60 mV s^−1^ between 0.4 V and +1.8 V using Bu_4_NClO_4_-MeCN as the electrolyte in a three-electrode electrochemical cell. As shown in Figure 4a, one pair of symmetrical redox peaks can be observed in the cyclic voltammetry (CV) curves, which presents excellent reversible electrochemical redox behavior. The oxidation peak is assignable to the Ru^2+^-to-Ru^3+^ conversion, and the oxidation potential correlates with the energy required to remove a d-electron from the Ru^2+^. Thus, the HOMO (the highest occupied molecular orbital) potential of metal ions is reduced, resulting in an increase in the bandgap of the MLCT, which can cause the MLCT behavior to no longer occur, as stated by M. Higuchi et al. [4]. The perchlorate ions are transferred from the electrolyte to the polyRu film to neutralize the change in the charge of the metal ions (from Ru^2+^ to Ru^3+^) during the oxidation process. Meanwhile, an exchange of the counter anion from Cl^−^ to ClO_4_^−^ in the electrode material occurs, as confirmed in Figure 3d. Additionally, the peak separation between the anodic and cathodic wave of the redox couple is not only related to the MLCT (metal–ligand charge transfer) transition energy but also to the ohmic drop [35,36,37]. Additionally, the peak current densities of the polyRu film increase with a gradual increase in the scan rate. The power–law relationship between peak current and scan rate can be calculated according to the following equation [38]:

(1) $$i=a^{v}\;b$$

in which both a and b are adjustable parameters, and i and v, respectively, represent the peak current and the scan rate. In general, b values of 0.5 and 1 indicate the kinetics of the redox reaction to be diffusion-controlled and capacitive-controlled process, respectively. As shown in Figure 4b, the b-values for the oxidation and reduction current peaks are, respectively, calculated to be 0.65 and 0.64, revealing that the current primarily arises from the pseudo-capacitive contribution in the redox system. Also, the high overlap of the two curves in Figure 4b further demonstrates the excellent electrochemical reversibility of the polyRu film. In addition, the proportion of diffusion behavior and capacitive behavior in the electrochemical redox process is calculated by the equation [39]: $Q\left(\nu \right)=Q_{c}+k\times \nu^{-1/2}$. As revealed in Figure 4c, the contribution of diffusion behavior occupies only a small part and gradually decreases from 15% to 6% as the scan rate increases from 5 mV s^−1^ to 60 mV s^−1^, indicating that the diffusion and exchange of counter ClO_4_^−^ anions only account for a small section and that most of the perchlorate ions act in the redox process through capacitive behaviors (containing surface, near-surface, and intercalation pseudo-capacitance). Therefore, according to the aforementioned morphological characteristics, the electrochemically cycled polyRu film possesses a large number of irregular nanopores due to the action of perchlorate ions. In situ Raman spectroscopic analysis is applied in order to further reveal the mechanism of anion-induced structural transformation and electrochromism of the polyRu film. Figure 4d shows the in situ Raman spectra of the polyRu film in different color and bleach states, which were monitored through cyclic voltammetry at a scan rate of 20 mV s^−1^ in 0.1 M Bu_4_NClO_4_-MeCN solution. The peak at 347 cm^−1^ originates from the metal–pyridine stretching mode [40]. The 404 and 665 cm^−1^ vibrations, respectively, involve the twisting and bending modes of the pyridine ring. The peaks at 848 cm^−1^ can be traced to the out-of-plane bending mode of the C-H bond [41]. In-plane bending deformations related to the C-H bond are seen at 1164 cm^−1^ and 1353 cm^−1^ [41]. The multiple intense peaks at 1014 cm^−1^, 1045 cm^−1^, 1237 cm^−1^, 1286 cm^−1^, 1468 cm^−1^, 1555 cm^−1^ and 1602 cm^−1^ are consistent with the pyridine ring stretching modes [42]. Obviously, there are no distinct differences in the Raman bands between the initial state and the reduced/oxidized state of the polyRu film, revealing that no chemical bonds are formed or broken in the polyRu film. In other words, most of the anions do not chemically react with the atoms on the electrodes, only undergoing charge transfer. Therefore, the electrochemical process of the polyRu film in the Bu_4_NClO_4_-MeCN electrolyte is highly reversible and rapid.

**Figure 5:**
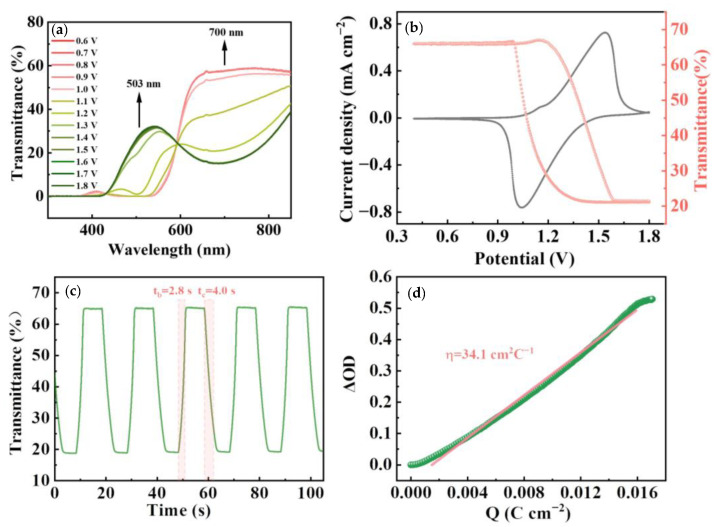
(**a**) In situ transmittance spectrum of the polyRu films at potentials from 0.6 V to 1.8 V. (**b**) CV curve and in situ transmittance changes at 700 nm of the polyRu film at a scan rate of 20 mV s^−1^. (**c**) Switching time and in situ transmittance spectrum at λ = 700 nm (0.4 V/+ 1.8 V, 20 s per cycle). (**d**) Variation in optical density (ΔOD) and charge density (Q) at λ = 700 nm.

Additional information can be obtained by investigating the effect of counter anions on the electrochromic performance of the polyRu film in a three-electrode electrochemical cell (Figure 5). Figure 5a displays the transmittance spectra of the polyRu film recorded at various applied voltages. Two distinct transmittance changes in the polyRu film in a wavelength range of around 503 nm to 700 nm are observed at an applied potential bias of 0.6 V to 1.8 V. The high coincidence of the transmittance curves at negative bias and forward bias suggests that the most effective electrochromic voltage window is between 1.0 V and 1.2 V. The initial observations suggest that there may be a stronger link between the color change in the polyRu film and the pseudocapacitive behavior of perchlorate ions than the number of ions embedded in the polyRu electrode. Therefore, the same maximum/minimum transmittance can be achieved under different negative/positive bias voltages, showing a fast response speed in Figure 5c (bleaching time t_b_ = 2.8 s, coloration time t_c_ = 4.0 s). And a large optical modulation of 42.4% can be achieved at 700 nm by applying an alternating potential between 1.2 V and 0.4 V. Figure 5b displays the in situ transmittance spectrum (at 700 nm) monitored by the CV at a 20 mV s^−1^ scan rate in the polyRu film. The transmittance is dynamically increased/decreased during the reduction/oxidative sweep process between 0.4 V and 1.8 V, which indicates a short switching time from rose red to dark green in the film (the insets in Figure 5b). In addition, as one of the essential parameters used to evaluate the electrochromic performance, coloration efficiency (η), defined as the change in optical density (ΔOD) caused by the charge stored per unit area of the polyRu film at a specific wavelength during the coloration process, can be calculated according the following equation [43]:

(2) $$\eta =\frac{\Delta \mathrm{OD}}{Q}=\log\;(T_{\mathrm{bleached}}/T_{\mathrm{colored}})/Q$$

where $T_{bleached}$ and $T_{colored}$, respectively, denote the transmittance of the film in bleached (reduction) and colored (oxidation) states. Q is the charge stored per unit area. Therefore, the coloration efficiency of 34.1 cm^2^ C^−1^ in the polyRu film can be calculated from the linear region of the curve in Figure 5d, which suggests rapid charge transport in the electrochromic film [23].

**Figure 6:**
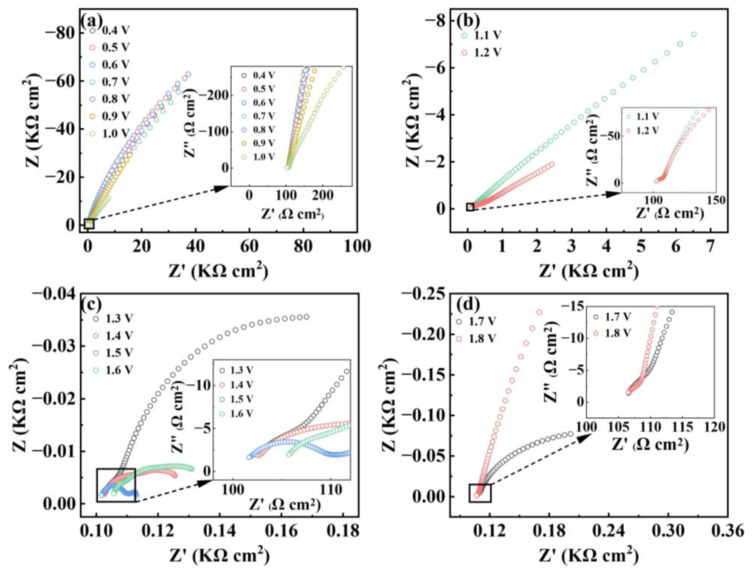
(**a**–**d**) Nyquist plots of the polyRu film at various constant potentials; the insets show the enlarged Nyquist plots in the high-frequency region.

Electrochemical impedance spectroscopy (EIS) measurements in the three-electrode cell were used to assess the kinetics of counter ClO_4_^−^ anions in the electrochemical process of the polyRu film. Figure 6a–d show Nyquist plots of EIS response for the polyRu film at various applied voltages. Commonly, a semicircle in the high-frequency region corresponds to charge-transfer processes, and an inclined line in the low-frequency region is known as the “Warburg region”, dominated by mass-transfer processes [27]. When the angle of the oblique line from the “Warburg region” is close to 45°, it is called “semi-infinite Warburg impedance” [44], while a linear line in the low-frequency region parallel to the Z” and Z’ axes represents purely capacitive and purely resistive behavior, respectively [45]. Electrolyte resistance (R_s_) and interfacial charge transfer resistance (R_ct_) can be simulated by the typical equivalent circuits [21] (Figure S3 and Table S1). As depicted in Figure 5a, in the range of 0.4–1.0 V, there is little variation in the EIS response with potential, with all Nyquist plots showing no noticeable semicircle (R_ct_ = infinite) at a high frequency, and then, a near-vertical capacitance region, indicating capacitive behavior of the film. Combined with the aforementioned transmittance spectra at different voltages, it can be seen that no redox reaction occurs (without interfacial charge transfer behavior) in the film, and the perchlorate ion (ClO_4_^−^) does not play a role in the voltage range of 0.4–1.0 V. On the other hand, the slope of the Nyquist plots at a high frequency declines and shows a semicircle when the voltage reaches 1.1 or 1.2 V, which can be attributed to the existence of interfacial charge transfer behavior at the electrode–electrolyte interface, revealing the occurrence of a redox reaction at the polyRu film interface accompanied by the diffusion and adsorption of the perchlorate ion (ClO_4_^−^). Further, the Nyquist plots display smaller semi-circle arcs, and the slopes of the linear lines in the low-frequency region are almost parallel to the Z’ axis when the voltage tends to be more positive (1.3–1.8 V), indicating low interfacial charge-transfer resistance (R_ct_) and purely resistive behavior of counter anion diffusion at the interface. The obtained results reveal that the high oxidation of the polyRu film (from Ru^2+^ to Ru^3+^) at the electrode–electrolyte interface under forward bias requires a large number of counter anions (ClO_4_^−^) in the electrolyte to move into and be stored in the polyRu film to balance the charge. Especially under a bias voltage of 1.5 V, two semi-circular arcs appeared at middle and high frequencies, originating from the ion storage processes of counter anions [45]. This result is consistent with the modulation of transmittance spectra upon forward bias voltages.

## 4. Conclusions

In summary, mechanistic insights into the anion-induced electrochromism of a Ru(II)-based metallo-supramolecular polymer are given in this paper. An evaluation of the kinetics of perchlorate ion (ClO_4_^−^) transfer and the coloration mechanisms depending on counter anion migration at the electrode–electrolyte interface is presented via in situ and ex situ analyses. An electrochemical process occurs via the capacitive behaviors of the perchlorate ions in which a containing surface, near-surface and intercalation pseudo-capacitance can be dominantly formed. The capacitive behavior of the perchlorate ions is responsible for the rapid switching speed of the polyRu film. Furthermore, the irregular nanopores induced by the perchlorate ion diffusion and the exchange of counter anions from Cl^−^ to ClO_4_^−^ in the film could be reasons for the degradation of the electrochromic performance of the polyRu film. More significantly, instead of being away from the film at negative bias, the perchlorate ions at positive bias diffuse and adsorb on the polyRu film to neutralize the change in the charge of the metal ions (from Ru^2+^ to Ru^3+^), accompanied by an exchange of the counter anions from Cl^−^ to ClO_4_^−^ in the film. Thus, part of the film material can be transformed from polyRu·(Cl^−^)_2n_ to polyRu·(ClO_4_^−^)_x_(Cl^−^)_2n−x_. This work opens new avenues for understanding the coloration mechanism and deepens the research on the kinetics mechanism of ion transport in metallo-supramolecular polymer films.

## Data Availability

The raw/processed data required to reproduce these findings cannot be shared at this time, as the data also form part of an ongoing study.

## Supplementary Materials

The following supporting information can be downloaded at: https://www.mdpi.com/article/10.3390/polym15244735/s1, Figure S1: A TEM image of polyRu; Figure S2: Cyclic voltammetry curves of the initial polyRu film and the polyRu films treated through CV after 100 and 1000 cycles; Figure S3: Stability over 5000 charge/discharge cycles; Figure S4: Digital photographs of the electrolyte before and after long-term cycling; Figure S5: The typical equivalent circuit used to fit the EIS spectra; Table S1: The value of parameters in the EIS spectra of the polyRu film at various potentials.
