# Supplementary material for: Mechanistic Insights into Anion-Induced Electrochromism of Ru(II)-Based Metallo-Supramolecular Polymer
Source: Polymers (Basel). 2023 Dec 18;15(24):4735. doi: 10.3390/polym15244735 (PMC10747135; doi:10.3390/polym15244735)
Supplement: Supplementary file 1 [file polymers-15-04735-s001.zip › polymers-2757912-supplementary.pdf]

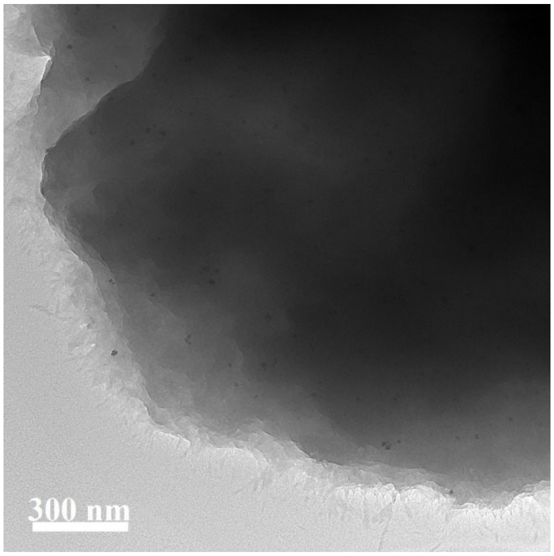

Figure S1. The TEM image of polyRu.

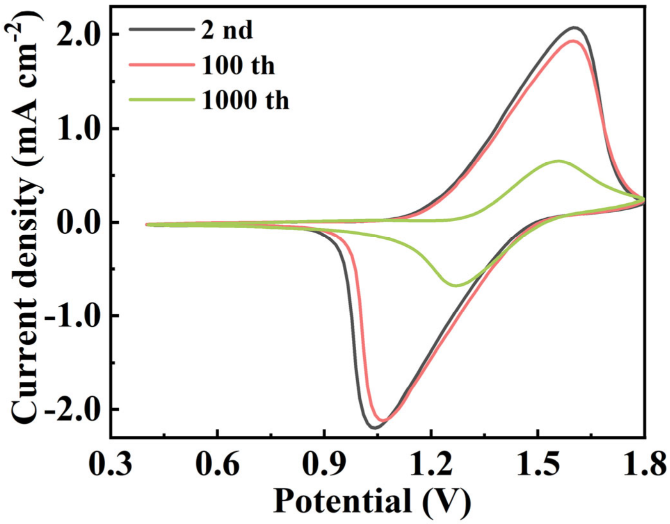

Figure S2. The cyclic voltammetry curves of the initial polyRu film and the polyRu films treated by CV after 100 and 1000 cycles.

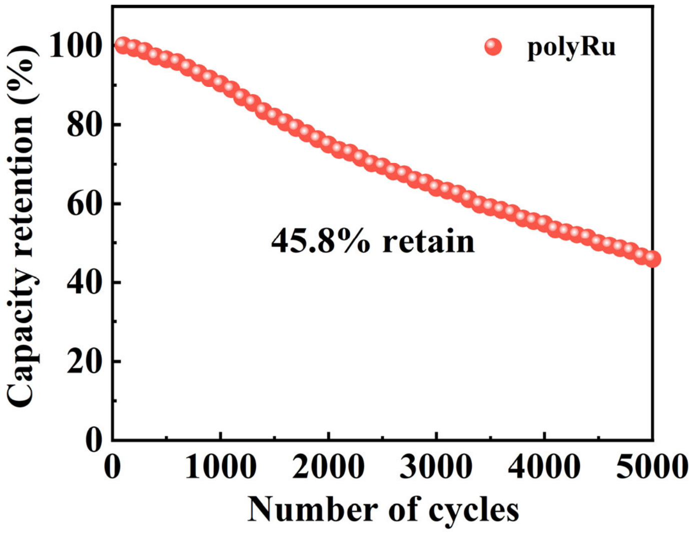

Figure S3. Stability over 5000 charge/discharge cycles.

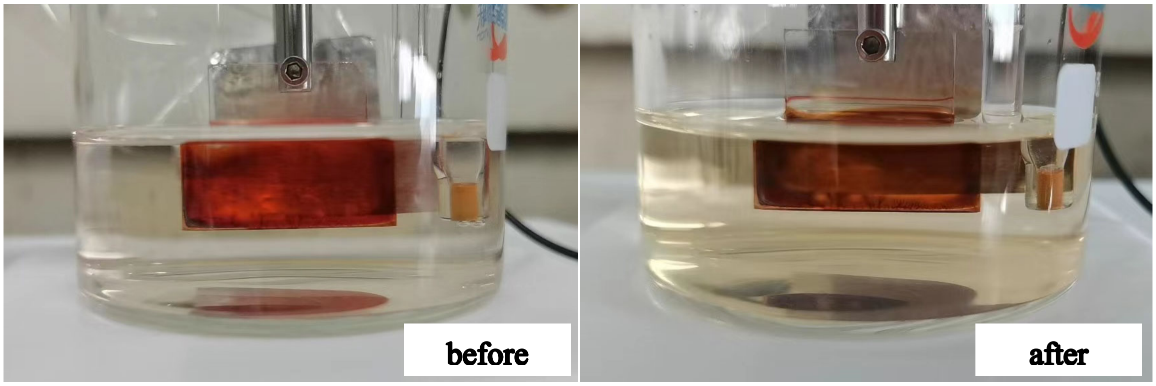

Figure S4. Digital photographs of the electrolyte before and after long-term cycling.

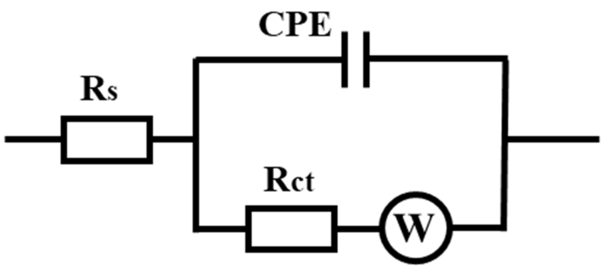

Figure S5. The typical equivalent circuit used to fit the EIS spectra.

Table S1. The value of parameters in EIS spectra of the polyRu film at various potentials.

Potential (V)	Rct (Ω)	Rs (Ω)
0.4	76.42k	41.91
0.5	81.85k	42.23
0.6	84.92k	42.22
0.7	29.80k	43.36
0.8	3.05k	42.70
0.9	2.61k	41.77
1.0	3.56k	41.42
1.1	399.20	41.80
1.2	141.10	42.63
1.3	29.25	41.60
1.4	7.67	37.41
1.5	4.57	41.25
1.6	7.92	41.54
1.7	79.59	43.43
1.8	824.40	44.32
